# Current Challenges Towards the Development of a Blood Test for Parkinson’s Disease

**DOI:** 10.3390/diagnostics4040153

**Published:** 2014-10-22

**Authors:** Jose A. Santiago, Judith A. Potashkin

**Affiliations:** The Cellular and Molecular Pharmacology Department, The Chicago Medical School, Rosalind Franklin University of Medicine and Science, North Chicago, IL 60064-3037, USA; E-Mail: jose.santiago@rosalindfranklin.edu

**Keywords:** biomarkers, blood, Parkinson’s disease

## Abstract

Parkinson’ disease (PD) is the second most prevalent neurodegenerative disease worldwide. To date, there is no disease-modifying agent, and current medical treatment only provides symptomatic benefits. Early diagnosis of PD would be useful in clinical practice to identify patients for clinical trials, test potential drugs and neuroprotective agents and track their therapeutic effect. Considerable progress has been made in the discovery and validation of diagnostic biomarkers for PD. In particular, blood-based biomarkers have shown promise in identifying PD patients in samples from independent clinical trials. Evaluation of these biomarkers in *de novo* patients and individuals at risk for PD remains a top priority. Here, we review the current advances and challenges toward the clinical translation of these biomarkers into a blood-based test for PD.

## 1. Introduction

Parkinson’s disease (PD) is a devastating neurodegenerative disease characterized by the progressive deterioration of the dopaminergic system in the substantia nigra pars compacta (SNpc). The prevalence of PD is estimated to be 1%–5% in individuals older than 50 years and rises steadily with a peak occurring between ages of 70 and 79 years [1]. Although the mechanisms underlying the disease pathogenesis remain elusive, a complex interaction between environmental stressors and genetic factors is believed to play a causative role in the disease. Accumulation of alpha synuclein protein (SNCA) is a central pathology of the disease, and recent findings have provided insights into the molecular mechanisms leading to SNCA aggregation, transmission and toxicity [2,3]. Current treatments improve symptoms associated with dopaminergic deficit, but advanced features of the disease, including falling, freezing and dementia, are not adequately controlled [4].

Diagnosis of PD is currently based on clinical assessment of motor symptoms. This notwithstanding, motor symptoms in PD patients are usually manifested later in the course of the disease, and by the time a patient is diagnosed, a substantial number of dopaminergic neurons are dead. Therefore, discovery and validation of accurate and sensitive biomarkers to identify individuals at early stages of the disease is expected to improve clinical management of PD. In this context, considerable progress has been made in the identification and validation of several molecular signatures associated with PD. In particular, blood-derived biomarkers are emerging as potential diagnostic tools for PD. Blood is an attractive source for biomarkers, because of its accessibility and the inherent biological and physiological information it can provide about disease status. Here, we summarize the most promising candidate blood biomarkers and the current challenges toward the translation of these biomarkers into the clinic.

## 2. Genetic Risk Factors as Blood Biomarkers for PD

PD is considered a sporadic disease with a prominent genetic component [5]. Several genetic risk factors have been identified in genome-wide association studies (GWAS), including mutations in the protein-coding genes, *SNCA*, *LRRK2*, *DJ-1*, *PARK2* and *PINK1*, among several others [5]. To date, the most extensively tested biomarkers in blood are those linked to a genetic mutation in PD. For instance, genetic triplication of the *SNCA* locus causes autosomal dominant PD, and the resulting increased expression of SNCA mRNA and protein can be detected in blood [6]. Thus, measuring SNCA protein in blood plasma was anticipated to provide a clinically relevant tool for PD diagnosis. Phosphorylated SNCA, but not total SNCA, was higher in blood plasma of PD patients at baseline compared to healthy controls [7]. The diagnostic performance of a biomarker is typically assessed by the area under the receiver operating characteristic curve (ROC). The area under the ROC curve for phosphorylated SNCA was 0.71 compared to 0.55 for total SNCA. This result showed a subtle improvement in the diagnostic accuracy values from the previously reported study of phosphorylated SNCA in blood of PD patients [8]. These findings suggested that phosphorylated SNCA is a potential biomarker for PD, whereas total SNCA had no diagnostic value [7]. Another study identified lower levels of endogenous SNCA antibodies in serum samples of PD patients compared to healthy controls, but did not achieve the required diagnostic criteria as a biomarker for PD [9].

Similarly, DJ-1 is another protein implicated in PD pathogenesis that has been evaluated as a potential biomarker for PD. The interest in DJ-1 as a biomarker for PD stems from the findings that mutations in *DJ-1* are associated with autosomal recessive early onset PD [10], and its antioxidant capacity makes it an attractive neuroprotective agent [11]. Post-translationally modified isoforms of DJ-1, but not total DJ-1 protein levels, were differentially expressed in blood plasma of late-stage PD patients [12]. However, accurate diagnosis of PD is challenging at early stages, thus the clinical utility of DJ-1 as a biomarker for PD is very limited.

Similarly, mutations in the leucine-rich repeat kinase 2 (*LRRK2*) gene are the most common cause of inherited PD and sporadic cases. Measurement of total LRRK2 or phosphorylated isoforms of LRRK2 were not differentially expressed in PD patients compared to healthy controls [13]. Collectively, these findings suggest that genes with mutations linked to PD do not necessarily reflect disease pathogenesis in blood, and protein biomarkers alone are not sufficient to diagnose PD patients accurately.

Nonetheless, some protein biomarkers have shown promise in identifying PD patients. For instance, a panel of 10 autoantibodies, including PTCD2, HSH2D, MYOT, EEF1A1, ICAM4, FRMD8, CTLA-4, PABPC3, FN1 and TRIM21, were capable of distinguishing PD patients from controls with 93.1% sensitivity and 100% specificity [14]. Several other protein biomarkers with direct biological implications in PD have been useful diagnostic tools. For example, plasma levels of 25-hydroxy-vitamin D_3_ protein were lower in PD patients compared to healthy controls, and its relative expression correlated with the Unified Parkinson’s Disease Rating Scale (UPDRS), a disease severity scale for PD [15]. These results reinforce the previously noted deficiency of vitamin D levels in PD patients [16]. In addition, levels of the antioxidant protein, glutathione S-transferase pi (GSTpi), were increased in blood of PD patients compared to healthy controls [17]. Inflammation has been largely implicated in the pathogenesis of PD [18,19]. In this context, PD patients had lower CD4^+^:CD8^+^ cell ratios and significantly increased ratios of IFN-γ-producing to IL-4-producing T-cells [20]. Thus, markers associated with vitamin D deficiency, oxidative stress and inflammation may be useful biomarkers for PD. The most important results obtained from protein blood biomarkers for PD are presented in Table 1.

**Table 1:** Candidate protein biomarkers in the blood of PD patients. The number of participants and the PD rating of the patients using the Hoehn and Yahr scale or the Unified Parkinson’s Disease Rating Scale (UPDRS) is provided under cohort characteristics. PD is Parkinson’s disease; HC is healthy control; AD is Alzheimer’s disease. N/A is not available because the results were not significant; AUC is area under the curve.

Biomarker	Description	Cohort characteristics	Assay	Diagnostic accuracy	References
SNCA	Phosphorylated SNCA was higher in blood of PD compared to HC.	PD: 189 Hoehn and Yahr: 1–2 HC: 91	ELISA	AUC = 0.72	[7,8]
DJ-1	DJ-1 isoforms were differentially expressed in late-stage PD patients compared to HC.	PD: 75 UPDRS <15: 15 UPDRS (15–30): 30 UPDRS >30: 30 HC: 30	Western blot	Not reported	[12]
LRRK2	Total LRRK2 or phosphorylated isoforms of LRRK2 were not differentially expressed in PD compared to HC.	PD: 33 HC: 27	Western blot	N/A	[13]
PTCD2, HSH2D, MYOT, EEF1A1, ICAM4, FRMD8, CTLA-4, PABPC3, FN1, and TRIM21	A panel of 10 autoantibodies identified PD patients from healthy controls and AD.	PD: 29 Early and late-stage PD HC: 40 AD: 50	Human protein microarrays	Sensitivity: 93.1% Specificity: 100%	[14]
25-hydroxy-vitamin D_3_	Levels of 25-hydroxy-vitamin D_3_ were lower in blood of PD patients compared to healthy controls and correlated with disease severity.	PD: 388 Hoehn and Yahr (mean): 2.1 UPDRS (mean): 31.1 HC: 283	Liquid chromatography/tandem mass spectrometry	N/A	[15]
Glutathione S-transferase pi (GSTpi)	Levels of GSTpi were lower in blood of PD patients compared to healthy controls.	PD:17 Hoehn and Yahr: 2 HC: 17	ELISA	Not reported	[17]

## 3. RNA Biomarkers for PD Identified by Microarray Gene Profiling

Unlike protein, RNA biomarkers have proven to be more sensitive and accurate in the diagnosis of PD. RNA is considered more advantageous, because very small quantities can be quantified by common biochemical assays, in particular by quantitative polymerase chain reactions (qPCR). Furthermore, RNA expression changes may reflect a response to environmental stressors, genetic factors and epigenetic changes through non-coding RNA.

The first investigations toward the discovery of blood RNA biomarkers for PD identified 22 differentially expressed genes that distinguished PD patients from healthy controls [21] and demonstrated that RNA expression changes in blood were useful to identify molecular pathways associated with PD and potential therapeutic targets [22]. The results from these studies suggested that cellular whole blood was an attractive source for PD biomarkers and rapidly prompted further investigations. Consequently, a molecular signature in blood composed of five genes, *SKP1A*, *HIP2*, *ALDH1A1*, *PSMC4* and *HSPA8*, classified early-stage and *de novo* PD individuals with 90.3% sensitivity and 89.1% specificity [23]. Two of these biomarkers, including *HSPA8* and *HIP2*, were confirmed by another microarray study; however, further confirmation by qPCR experiments was not performed [24]. 

In earlier studies, total mRNA abundance was measured by standard microarrays. However, more than 90% of the human pre-mRNAs are alternatively spliced [25]; therefore, many splice variants may have been missed in these studies. In addition, alternative splicing responds to environmental factors, thus changes in pre-mRNA splicing in the blood of patients was anticipated to provide insights into the disease pathogenesis and a rich source of biomarkers for PD. Analysis of previous microarray data identified the splicing factor, serine/arginine repetitive matrix 2 (*SRRM2*), dysregulated in the blood of PD patients compared to healthy controls [26]. Furthermore, splice variant-specific microarrays were used to screen RNA prepared from whole blood of PD patients, atypical parkinsonian disorders (APD) and healthy controls. A molecular signature composed of 13 splice variant biomarkers, including *C5ORF4*, *COPZ1*, *MACF1*, *WLS*, *PRG3*, *ZNF160*, *EFTUD2*, *MAP4K1*, *MPP1, PKM2*, *SLC14A1-s*, *SLC14A1-l* and *ZNF134*, distinguished PD patients from APD and healthy controls with 90% sensitivity and 94% specificity [27]. Seven out of the 13 splice variant biomarkers, including *C5ORF4*, *COPZ1*, *MACF1*, *WLS, PRG3*, *ZNF160* and *EFTUD2*, were replicated in blood samples obtained from an independent clinical trial, thus strengthening the association of aberrant splicing in PD [28]. These biomarkers are currently being evaluated in blood samples from non-medicated patients and individuals at risk of PD. Furthermore, exon arrays revealed several alternative splicing aberrations in the blood of PD compared to healthy controls [29]. More recently, analysis of blood cell transcripts by RNA-sequencing has been demonstrated to be useful to identify long-non-coding RNA and alternative splicing events characteristic of PD [30].

## 4. Network-Based Biomarkers for PD

Microarray and high-throughput technologies for gene expression have been essential to identify differential patterns of gene expression characteristic of disease. Nonetheless, the interpretation of such gene expression patterns can be difficult. Network biology provides a template to understand the molecular interconnections and biological pathways associated with disease while offering computational tools to prioritize and identify biomarkers and therapeutic targets for neurodegenerative diseases [31].

Network-based approaches have shown promise in the discovery and prioritization of biomarkers for PD and atypical parkinsonian disorders. Given the clinical overlap between PD and several atypical parkinsonian disorders, there is a high misdiagnosis rate at early stages of the disease. For instance, progressive supranuclear palsy (PSP) patients may be misdiagnosed with PD. Recently, a network approach identified a molecular network of 843 genes closely connected to genetic risk factors associated with PSP [32]. Network prioritization identified the protein, tyrosine phosphatase, non-receptor type 1 (*PTPN1*), as a highly ranked gene within the functional linkage network associated with PSP. Evaluation of *PTPN1* mRNA in blood samples by qPCR assays revealed that it was useful for distinguishing PSP from PD patients with 84% sensitivity and 73% specificity [32]. Validation of *PTPN1* mRNA in an independent set of samples will be important to assess its clinical utility as a diagnostic tool for PSP.

Network biology approaches have been applied to study the impact of disease comorbidities associated with PD and to identify potential biomarkers. Epidemiological evidence suggests type 2 diabetes (T2DM) is a risk factor for PD [33,34,35,36,37], reviewed in [38]. It has been proposed that disruption in shared biological pathways, including impaired insulin signaling, mitochondrial dysfunction and endoplasmic reticulum stress, may lead to both chronic diseases [38,39]. Several system-biology approaches have provided insights into the molecular mechanisms underlying the relationship between PD and T2DM and potential therapeutic targets [40].

In this context, a network-based approach to decipher the association between PD and T2DM, identified a network of 478 genes shared between known risk factors for T2DM and PD [41]. Combinatorial network analysis with microarray studies identified the amyloid precursor protein (*APP*) as a highly ranked gene within the network. *APP* mRNA achieved a diagnostic accuracy of 80% in distinguishing PD patients from healthy individuals in blood samples obtained from two independent clinical trials [41]. The role of *APP* in PD remains unknown, but several studies have revealed possible mechanisms by which the protein may affect disease onset or progression. *APP* is a precursor protein that undergoes proteolysis to produce beta amyloid peptides (Aβ). The amyloid fibrillar form is found in the amyloid plaques of Alzheimer’s disease patients (AD). With regards to Parkinsonism, Aβ promoted the aggregation of SNCA in a transgenic mouse model [42]. In addition, expression levels of Aβ peptides in cerebrospinal fluid (CSF) were associated with motor deficits in PD patients [43].

More recently, network analysis of shared genetic connections between PD and T2DM obtained from disease-gene databases that included environmental factors identified the superoxide dismutase 2 (SOD2) as a potential biomarker for PD. Relative abundance of *SOD2* mRNA was upregulated in the blood of PD patients compared to healthy controls in samples obtained from two independent clinical trials [44]. Given that T2DM is associated with the worsening of motor symptoms in PD [35,45], it is plausible that some of the shared genes between PD and T2DM may be indicators of disease severity and/or progression of PD. 

The most promising results from blood RNA biomarkers for PD are shown in Table 2. The results from the above studies should be interpreted with caution, as further evaluation of these biomarkers in samples from drug-naive patients and at-risk individuals in larger longitudinal studies will be essential to conclusively validate their clinical utility. With the increasing gene expression data deposited in publicly available databases, network-based approaches provide an amenable tool for the discovery of potential biomarkers and therapeutic targets for PD and related parkinsonian disorders.

**Table 2:** Candidate RNA biomarkers in the blood of PD patients. The number of participants and the PD rating of the patients using the Hoehn and Yahr scale or the Unified Parkinson’s Disease Rating Scale (UPDRS) is provided under cohort characteristics. PD is Parkinson’s disease; HC is healthy control; AD is Alzheimer’s disease; MSA is multiple system atrophy; PSP is progressive supranuclear palsy; AUC is area under the curve.

Biomarker	Description	Cohort characteristics	Assay	Diagnostic accuracy	References
*SKP1A, HIP2, ALDH1A1, PSMC4, HSPA8*	A five-gene panel distinguished early-stage and *de novo* PD individuals from HC.	PD: 92 Hoehn and Yahr (mean): 1.9 HC: 64 AD: 29	qPCR	Sensitivity: 90.3% Specificity: 89.1%	[23]
*C5ORF4, COPZ1, MACF1, WLS, PRG3, ZNF160, EFTUD2, MAP4K1, MPP1, PKM2, SLC14A1-s, SLC14A1-l, ZNF134*	Thirteen splice variants distinguished PD from HC and APD patients.	PD: 51 Hoehn and Yahr: 2 HC: 21 PSP: 17 MSA: 17	qPCR	Sensitivity: 90% Specificity: 94%	[27]
*C5ORF4, COPZ1, WLS, PRG3, ZNF160, MACF1, EFTUD2*	Seven splice variants were replicated in the HBS study and distinguished PD from HC.	PD: 50 Hoehn and Yahr (mean): 1.9 HC: 46	qPCR	Sensitivity: 78% Specificity: 90%	[28]
*APP*	*APP* mRNA distinguished PD patients from HC in two independent cohorts of patients.	PD: 101 Hoehn and Yahr (mean): 1.9 HC: 91	qPCR	Sensitivity: 80% Specificity: 60%	[41]
*SOD2*	Relative abundance of *SOD2* *mRNA* was upregulated in PD patients compared to HC.	PD: 101 Hoehn and Yahr (mean): 1.9 HC: 91	qPCR	AUC: 0.69	[44]

## 5. Current Challenges towards a Diagnostic Tool for PD

Although substantial progress has been made in the discovery of potential blood biomarkers for PD, there remain a few limiting factors hampering the translation of these biomarkers into the clinic. Good practices for RNA biomarkers in PD include the evaluation of RNA purity and mRNA integrity, ascertainment of PD diagnosis and controls, the inclusion of *de novo* PD patients, adjustment for known risk factors and independent validation [46]. In this regard, most of the proposed blood molecular signatures for PD are yet to be replicated in samples from independent populations. Another consideration is the evaluation of each biomarker in samples from non-medicated patients to determine whether and to what extent medication affects gene expression. Moreover, replication in larger prospective studies will be crucial to determine their clinical utility and to identify potential biomarkers to monitor disease progression and severity. Ideally, evaluation of biomarkers in samples from patients at risk of the disease will be key to determine whether biomarkers can predictably identify patients with asymptomatic PD.

To address these challenges, several ongoing clinical trials are actively recruiting participants and collecting biological samples, including RNA isolated from cellular whole blood that is available to researchers. The Harvard Biomarker Study (HBS) is a longitudinal case-control study of over 2100 patients with early-stage PD and controls without neurological disease [47]. Clinical information about study participants and availability of biological samples can be found elsewhere in [48]. Several studies using samples from this cohort of patients have been published recently [28,41,47,49]. Similarly, the Parkinson Progression Marker Initiative (PPMI) is an observational, international and multi-center collaborative study designed to identify biomarkers to monitor PD progression. This cohort of participants contains over 400 non-medicated patients and 200 healthy subjects [50] from which blood, cerebrospinal fluid and urine samples have been collected over several years. More information about the study and sample availability can be found at [51].

An important milestone in the search for biomarkers for PD is to identify those patients at risk for the disease. The onset of impaired olfactory function in PD patients typically precedes motor symptoms and could be used as a screening test for patients at risk of PD. For this purpose, the Parkinson At-Risk Syndrome Study (PARS) is designed to identify biomarkers that can predictably identify individuals at risk of PD using prodromal non-motor features along with olfactory testing [52,53]. More information about how you can participate in this study can be found at [54].

## 6. Conclusions

There is a steady increase in efforts to identify and validate diagnostic tools for PD. Blood-based biomarkers from RNA hold great promise because of the high sensitivity and specificity achieved in recent studies. In addition, the protein biomarker 25-hydroxy-vitamin D_3_ may be useful as a disease severity biomarker and to evaluate disease progression in PD. Network-biology approaches are emerging as a valuable tool to identify highly accurate and biologically relevant biomarkers for PD. In this regard, analysis of disease comorbidities associated with PD, for example, diabetes, may reveal important biomarkers and therapeutic targets.

Biomarkers are needed to evaluate the therapeutic effect of potential drugs and neuroprotective agents that are evaluated in clinical trials. For example, common drugs prescribed to treat patients with diabetes, such as exenatide, have been shown to improve motor symptoms in PD patients [55,56,57]. Likewise, thiazolidinediones, a group of PPAR-**γ** agonists, are currently being tested as potential disease-modifying agents for PD [58]. Currently, these studies examine motor symptoms to determine the efficacy of the agent being tested. In the future, it would be advantageous to have reliable sensitive biomarkers that can be used in clinical trials so that subtle improvements in PD patients could be monitored. Future directions in the quest for a diagnostic tool for PD include the replication of RNA blood-based biomarkers in samples from non-medicated patients (PPMI) and individuals at risk of PD (PARS).
